# Book Reviews

**Published:** 1819-12

**Authors:** 


### CRITICAL ANALYSIS

OF

### RECENT PUBLICATIONS, IN THE DIFFERENT BRANCHES OF MEDICINE AND SURGERY.

"I would have men know, that, though I reprehend the easie passing over of the causes of things, "by ascribing them to secret and hidden vertues and properties; (for this hath arrested and laid "asleepe all true enquiry and indications;) yet I doe not understand but that, in the practical "part of knowledge, much will be left to experience and probation, whereunto indication cannot "so fully reach: and this not only in *specie*, but in *individuo*. Yet it was well said, *Vere scire* "*esse per causas scire*."—BACON.

*Medico-Chirurgical Transactions, published by the Medical and Chirurgical Society of London.* Vol. IX. Part II. Longman and Co. London. 1818.

THIS Part commences with some histories of *Cases of Fungus Hæmatodes, Cancer, and Tuberculated Sarcoma, with Observations*, by GEORGE LANGSTAFF, Esq. possessing the highest degree of interest; though we must acknowledge, that this has probably been rendered more apparent to us, from our having previously perused the work of Dr. BARON. We, however, regret that the arrangement made of the subjects engaging our attention had caused us to defer until the present time the perusal of the observations of Mr. Langstaff; for, had we been acquainted with them before the analysis of Dr. Baron's treatise was made, we should not only have illustrated it by the coincident observations and opinions here alluded to, but have also shown more disposition to the admission of his theory than we then evinced. Yet Mr. Langstaff had previously advanced similar observations, and a glimpse of similar theoretical views, in a paper published in the eighth volume of the present series of Transactions: but they were but little more than a blank to us, and, we must add, to the greater number of pathologists. This is a fact which forcibly shows how important it is that persons, pursuing any particular series of researches, should publish, with their observations, their theoretical views; for, without these, the importance of many facts must be lost to persons in general, who have not devoted much time to reflection on the same subject. Even hypotheses may not be devoid of utility on these occasions; for, though they suppose relations between phenomena that may not exist, they may point out others that are real; and this consideration should deter men from too prompt a disposition to censure them, even when erroneous, if produced with so laudable and legitimate a view, and if given with proper reserve.

In the observations here related, and in the opinions that cancer, fungus hæmatodes, medullary and tuberculated sarcoma, have the same origin, Mr. Langstaff agrees with Dr. Baron; with this exception only, that the former pathologist has not traced those morbid states from the hydatid or simple cyst in so determinate a manner; nor has he apparently so far generalized his views. In order to show this, we shall first adduce his general conclusions, and then some of the most interesting of his particular observations and reflections. Mr. Langstaff says,

"In the history of the preceding cases, I have endeavoured to show that there exists a close resemblance in the morbid structure of fungus hæmatodes, pulpy or medullary sarcoma, tuberculated sarcoma, and carcinoma; or that those diseases may exist together in the same person, locally and constitutionally; or that fungous tumors may become cancerous, as, Sir Everard Home supposes, any kind of tumor may."

And Mr. Langstaff, in a note, makes a remark, that must not be disregarded in reflecting on the above:

"Scrofulous patients (Mr. L. says) likewise frequently die of tubercular phthisis, after having suffered amputation of an arm or a leg: they are also liable afterwards to attacks of struma in the joints; for I have, on dissection, in those subjects, found the bones forming the principal articulations much softer than natural, and a caseous deposition in the interstices of the cancelli."

From having observed fungus hæmatodes in every part of the body, that is, in every organ except the nerves, tendons, and cartilages, Mr. Langstaff is disposed, from its general distribution, to consider the cellular membrane as the seat of the disease; or, at least, that it is the part on which those morbid changes are deposited, or from which they are produced.

The same affection has occurred to the author's observation in monkeys, horses, oxen, sheep, pigs, dogs, cats, and birds. Monkeys are very subject to scrofula, he continues to remark; and, in a great number of those brought to this country which he has examined, he has generally found that they have died of scrofulous tumors, partly in the state of vomicæ. Mr. Langstaff says, he does not think that these abscesses are produced by the suppuration of tubercles, but by affection of the healthy surrounding structure, caused by the irritation of the tumors. But, in opposition to this remark, it should be observed, that suppuration generally [from our own observation, we would say almost always,] begins in the centre of the tubercles.

Mr. Langstaff has also observed, that

"Sometimes the schirrous, medullary, and fungous, structures, are so blended in various parts of the same subject, that they appear like different stages of morbid growth; and the difficulty which the pathologist experiences, is in deciding whether the disease of the cancerous or hæmatodal kind, or whether they are not of the same class. Sir

Everard Home, in his observations on the nature and progress of Cancer, says, so much does the same disease differ in its appearances in different patients, from the endless peculiarities of their constitutions, by which every part of the body must be more or less influenced, that it is not possible, in practice, to distinguish in all cases between cancerous and scrofulous tumors, after they have advanced to a certain size. He is ready to confess that, in many instances, he has mistaken the one for the other; and has removed by operation tumors which, at the time, had the appearance of being cancerous, and, upon examination after their removal, found them of a scrofulous nature. On the other hand, he has neglected to remove tumors, from the circumstances making it probable that they were scrofulous, which afterwards became cancerous, and destroyed the patient."

In most of these general notions, Mr. Langstaff agrees with Dr. Baron; and, in the following particular remark, he also favours his views of the important connexion of these affections, in some instances at least, with morbid action of the absorbent vessels. In noticing a case of this disease in the liver, Mr. Langstaff says, "The tumors were not enveloped by capsules, but so intimately connected with the remaining structure of the liver, as to induce me to suppose there must have been first interstitial absorption, to allow the vessels to take on the office of depositing the diseased masses." This, however, opposes the notion of the hydatidical origin of the disease. On many other occasions, however, the author has observed that the tumors "possessed delicate cysts."

Mr. Langstaff also witnessed several cases where a bloody fungus, precisely similar in appearance to that supposed to mark a particular disease which has thence received its name, has arisen at a late stage of the organic lesion: in some instances, from spontaneous change; in others, from the parts having either been handled or cut into by the knife: and he is inclined, as well as Dr. Baron, to believe that this fungus is only one of the effects of the common disease. The author adduces many particular observations, supporting his opinion respecting the seat of the disease: in the lungs, the tumors occupied "chiefly the surface beneath the pleura, many of which had, by their growth, occasioned the absorption of that membrane, which allowed of their growing into the cavity of the chest;" many of them, although enveloped with a cyst, formed by the pleura, projected considerably from the lungs. Similar appearances were observed between the layers of the omentum, and beneath the peritoneal covering of the intestines. The following observations, besides corroborating the opinion above alluded to, furnish some interesting facts respecting the progress of the disease:

"Some of the tubercles were very small; the largest were near the surface of the liver, and none exceeded the magnitude of the common walnut. They all had delicate cysts, and the contained matter was retained by beautiful reticulated lymph. The large kind were formed by an aggregation of the smaller: they did not project far from the surface of the liver, but had a slight central depression, without the peritoneal covering being thickened; but the blood-vessels were very numerous in those parts, and most likely would, if the man had lived much longer, have occasioned that cartilaginous opacity mentioned in some of the former cases."

These coincidences of opinions in different pathologists are highly gratifying; and the circumstance of their having occurred in Dr. Baron and Mr. Langstaff, without any mutual intimation of sentiment, adds much to the weight of each individually.

We have indulged in many of the foregoing extracts, because they will supply a deficiency in our analysis of the work of Dr. Baron; for his and Mr. Langstaff's observations of facts so nearly agree, that it is unimportant from which they are taken: but we cannot extend them much further. The following, however, we adduce, as being of great importance in a therapeutical point of view; since, with the fact that the disease when it appears in the extremities is but very rarely confined to them, it must show the impropriety of adding to the distress of the patient, in a great proportion of cases, by operations with the knife, as frequently as we have been accustomed to do.

"From what I have seen (says Mr. Langstaff) of cancer and fungus hæmatodes, I am convinced of their not being local diseases, any more than those of the scrofulous class: they must, therefore, be considered what has been termed constitutional. And, having noticed many small scrofulous tubercles in the lungs of still-born children; also pulpy tumors in the lungs of adult persons who had not been affected during their lives with the least symptom of pulmonic disorder, and who died of active disease of a different description in other viscera; I am led to suppose, that these specific and malignant diseases, such as cancer, fungus hæmatodes, and scrofula, have their origin perhaps with the formation or development of the natural parts of the fœtus in utero; and that they remain, after the birth of the individual, in some instances, dormant or inactive for a series of years; and, in all, only require a peculiar morbid excitement, to occasion their increase and destructiveness."

Before we conclude, we are disposed to make a remark respecting the multitude of cases of this disease which have occurred to the observation of Mr. Langstaff, and which are so far beyond the proportion witnessed by other surgeons in extensive practice; it may probably be thus accounted for: Mr. Langstaff is surgeon to the workhouse of the ward of Cripplegate Without, which generally contains upwards of 270 paupers, and of the most wretched kind; "many of whom are admitted with the worst species of disease, besides having suffered, previous to their admission, all the privations which abject poverty occasions." It was there the author witnessed a great proportion of the cases here related: and this is worthy of consideration, in connexion with the facts mentioned in Dr. Baron's work, of Dr. Jenner having found that he could produce tubercles in young animals by feeding them on certain species of food, and especially by not allowing them a sufficient quantity of nutriment. Let us add to this a remark of Dr. Baron, the importance of which has now more fully struck us,—that, a peculiar want of nutrition marks the earliest stage of the apparent existence of the tuberculous diseases he has described.

All those who are but half as deeply impressed with the importance of this subject as we are, will make it an object for meditation, after having become acquainted with the facts respecting it, to which we have alluded, by the perusal of the original works.

*Case of Hydrocephalus, successfully treated by the removal of the Water by Operation. By* JAMES VOSE, M.D. of Liverpool.

The patient was an infant, seven weeks of age, whose head was enlarged to between two and three times its natural size. Ossification had made little progress since birth: shortly after which, the existence of the disease was observed. The head, when placed betwixt the eye and the light, was so transparent as to be not unaptly compared to a paper-lantern.

The operation was performed by a couching-needle, but it is neglected to be stated in what part the puncture was made. Three ounces and five drachms of limpid fluid were discharged, and the opening was closed with adhesive plaster; a roller at the same time being applied round the head. The head lost its tension and globular form, allowing the water to gravitate with the motion of the now flaccid parietes. About the same quantity of water dribbled from the orifice after the operation; and the child sunk so low as to create great alarm. It rallied, however, without the aid of medicine; and water again accumulated in a few days, so as to produce the former degree of tension. The operation was repeated in about a fortnight, with less fear and circumspection. A bistoury was used, and five ounces of fluid were evacuated. No unpleasant consequences ensued.

The head, having regained its former size in three weeks' time, was again tapped, and eight ounces of fluid abstracted, without the production of any constitutional disturbance. The head was punctured, for the last time, about nine days subsequently; and a grooved director being introduced into the orifice, twelve ounces were drawn off in a continued stream.

The head became very flaccid and shapeless, but no derangement of health followed. It was observed that, after each operation, the process of ossification went on with great rapidity.

Shortly after the last operation, a copious discharge of water from the bowels occurred; at first with the natural motions, but afterwards alone, and resembling in sensible qualities that evacuated from the head. The same sinking as followed the first puncture ensued on the second day of this discharge; and it was remarked, that a diminution of the size of the head corresponded with the quantity of fluid thus discharged. Ossification advanced rapidly; and, at the time of the report, the bones of the head were nearly as complete as is usual in a child of that age. The infant improved in health, size, and vigour: its appetite and digestion were good; and not a single convulsion had occurred since the first operation. The medical treatment was confined to a regulation of the bowels by small doses of hydrarg. cum creta.

The importance of this case will, we trust, warrant this ample detail. A want of the previous history of the disease must be regretted.

As we are about to prepare a review of the Essay of Dr. Cheyne on Hydrocephalus, we shall defer our remarks on this case. In that, we shall give an account of another instance in which a similar operation has lately been performed with success, and show that it was advised as a common practice by Hippocrates.

*Account of a Case of defective Power to distinguish Colours.* By Whitlock Nicholl, M.D. F.L.S. of Ludlow. Read May 26, 1818.

This case is similar to one before related by Dr. Nicholl, and published in the seventh volume of these Transactions.

*On the Use of the Actual Cautery as a Remedy for the Cure of Diseases.* By J. P. Maunoir, Professor of Surgery in the University of Geneva.

*Further Account of the result of an Operation for forming an artificial Pupil. Extracted from Letters addressed to Professor Scarpa.* By the same.

This will not admit of an abstract.

*Cases shewing the Coincidence of Worms in the Intestines with Hæmoptysis, and Remarks on the Probability of the two Affections having a Connexion with each other.* By Nathaniel Rumsey, Esq. Surgeon, Beaconsfield, Bucks. Communicated by Dr. Bateman. Read June 24, 1818.

The connexion of hæmoptysis with a disordered state of the intestinal canal, has been frequently observed; and those physicians who have most attended to verminous affections, have been led to believe that their presence in the bowels is more frequently a consequence than a cause of a disordered state of those viscera. This indication is almost solely followed by the Italians in their treatment of those maladies; and every person who has witnessed their practice, is forcibly struck with their extraordinary success. We must however remark, respecting this paper, that Mr. Rumsey's observations have led him to think that intestinal worms derive their origin from some morbid secretion into that canal, not from *ova*. In this opinion he will find himself very powerfully supported by RUDOLPHI, whose accurate researches on this subject have determined the question, in the judgment of the greater number of pathologists on the continent.*

*Two Cases of Aneurism, in which the Temporary Ligature was employed.* By B. TRAVERS, Esq. F.R.S. Vice-President of the Society. Read May 26, 1818.

The ligature was employed, in the first, for aneurism of the radial artery below the elbow, by a loop-knot, and was removed fifty hours afterwards. Obliteration of the artery above the situation of the ligature was effected. But, from the circumstances which occurred in the second case, Mr. Travers expresses his determination to relinquish the use of the temporary ligature; and, he adds, he should regret to hear of the repetition of the experiment. The grounds of his expectation, and the motives which led him to the practice, will be seen in his papers in the fourth and sixth volumes of the Society's Transactions. He candidly acknowledges that he had formed too favourable an opinion of it; but that it is to him "a source of high satisfaction, that the decision of the question, which I deemed of importance, has been obtained without loss of life or limb."

The use of a cylinder of waxed linen, to be placed between the artery and the ligature, which Mr. Travers speaks of in this paper, though not in a favourable manner, as a means not so likely to be followed by secondary hæmorrhage as the simple ligature, has been noticed in the 245th volume of this Journal. The most important objection is, we think, the extensive separation of the artery from the surrounding parts which it requires; a thing itself likely to be followed by ulceration of the artery, and consequent hæmorrhage. Yet SCARPA still favours this practice. The question respecting its utility cannot be considered finally determined.

Mr. Travers says, he has long been in the occasional practice of applying the noose-ligature to the arteries of stumps, and

* See *Entzoorum Vermium Intestinalium Historia Naturalis.* Auctore C. A. RUDOLPHI. Amstelodamiæ, 1808, 1810, and 1819.

removing them at the first dressing; and that he has not known a single instance of secondary hæmorrhage, after the use of that measure.

*Observations on some Points relating to the Physiology and Pathology of the Ear.* By Joseph Swan, Esq. Surgeon of Lincoln Hospital. Read May 12, 1818.

We have transcribed this little memoir into our *Collectanea.* As it is only an exposition of observations, it does not admit of any other critical comments than such as designate the interesting nature of the facts detailed.

*Account of a Case in which some singular Preternatural Appearances were observed in the Ovarium and Female Bladder.* By Edward Phillips, M.D. of Andover. Read June 24, 1818.

The interest of this case consists in the singular appearances witnessed after death: of these we shall transcribe the history. The symptoms during life, were only those of irritation of the bladder two years previously to the fatal termination of the malady; and, soon afterwards, all those which generally accompany the more active forms of disease of the ovaries. Yet it is singular, that, when the signs of the disease of the ovary became more forcibly characterized, all the symptoms of irritation of the bladder were suspended, and continued so, Dr. Phillips remarks, until the patient was released from her sufferings. The patient was about thirty years of age; but the author has omitted to mention whether or not she was married.

" *Dissection.*—The body was opened fourteen hours after death, by Mr. Pitman, jun. a young surgeon of great promise; and the following particulars were the result of the examination:

" On opening the cavity of the abdomen, there escaped about two gallons of water mixed with blood. On the left side of the umbilical region there was an ovarial tumor, rather larger than a human heart, the contents of which were a semi-fluid substance, a good deal resembling in appearance clouted cream. In the middle of this cream-like substance was found a tuft of hair, about the size of a hen's egg. The surface of the tumor was nearly covered by clusters of hydatids, beautifully transparent, and connected like a bunch of grapes. On the broad ligament of the left side of the uterus there arose a number of small white tumors, of the size of common peas. The uterus itself was not diseased. The bladder was very much distended, or rather plugged-up, with a substance similar to that which was contained in the ovarial tumor; and here also was discovered another large tuft of hair. The coats of the bladder were very much indurated, and particularly the inner coat. The urethra appeared to have no direct communication with the bladder, at the under and posterior part of which there was attached a small cyst, having the same cream-like substance before described, and also a quantity of hair; and, what is deserving of particular notice, there was a perfectly-formed incisor tooth, having the enamel and its fang firmly attached to the coats of the cyst.* This cyst or cavity had a communication with the bladder by means of three small foramina, and it communicated also with the urethra on the anterior part."

*An Account of a Congenital Monstrosity.* By G. BRESCHET, M.D. Prosector of the Faculty of Medicine at Paris, and Chief of the Clinical Department at the Hôtel-Dieu, &c. Read June 24, 1818.

We shall give the author's recapitulation of the remarkable circumstances presented on examination.

"1. Curvature from rickets; the lower extremities turned back, so as to correspond with the posterior part of the trunk.

"2. The placenta applied to the abdomen.

"3. The membranes of the ovum contributing to complete the anterior side of the abdomen.

"4. The absence of the umbilical cord, or, to speak with more precision, the vessels which usually compose this cord not twisted together; one artery, with the vein, passing by the left hypochondrium to the upper surface of the liver, and thence proceeding to its concave surface.

"5. The absence of the kidney on the right side.

"6. The absence of ovarium and fallopian tube on the same side.

"7. The separation of the os pubis, and retroversion of the right os ischium.

"8. The bladder appearing externally, and presenting only its posterior surface, on which the orifice of the left ureter could be seen.

"9. The anus situated a little underneath this orifice of the ureter.

"10. The obliteration of the vagina.

"11. A spina bifida, or destruction on the right side of the processes of the last lumbar vertebræ, and of the posterior side of the spinal canal of the os sacrum.

"12. The total absence of nerves and muscles of the whole of the right inferior extremity, and their place being occupied with a species of solid fat.

"13. Deficient development of the bones of the same limb."

*On the Comparative Infrequency of Urinary Calculi among Seafaring People.* By A. COPLAND HUTCHISON, Esq. Surgeon Extraordinary to his Royal Highness the Duke of Clarence, Surgeon to the Westminster General Dispensary, and late Surgeon to the Royal Naval Hospital at Deal. Read May 12, 1818.

This memoir is drawn up with the intention to furnish precise information respecting one part of the subject of calculous disorders, which Dr. Marcet, in his essay, has left open for the

* "The preparation exhibiting these appearances, is deposited in the Museum of the College of Surgeons. On examining it more accurately, it was found that the tooth was imbedded in a portion of bone resembling an alveolar process."

contribution of future discoveries. The researches on which the statements in it are founded, have been made with the utmost care, and on so comprehensive a scale, as to render the view of this part of the enquiry complete. The utility of the observations with respect to medicine, cannot be at all estimated in the present state of our knowledge; but, as a statistical record, this memoir must be considered highly valuable.

Calculous disorders have ordinarily been considered as especially within the domain of chemistry; and an attempt has been recently made, by an eminent physiologist, to render the medical treatment of them subservient to the principles of that branch of science. Being decided enemies to such views, we have regarded the observations and reflections of Mr. Hutchison with a degree of pleasure that the partizans of the doctrine just alluded to would probably consider malicious: an abstract of them will show on what foundation.

During a period of sixteen years, from 1800 to 1815 inclusive, about 162,000 men have been annually voted by Parliament to man the British navy: the annual new supply has been, on an average, 30,000; but of these men, nine-tenths had served at sea from a very early period of life. The number of patients (exclusive of soldiers, Russians, and prisoners of war,) admitted into the Naval Hospitals of Haslar, Plymouth, and Deal, during the same period, was 96,697: amongst these there were only *eight cases* of stone in the bladder; and, even of these, two were boys who had laboured under symptoms of that affection for some years previously to their entry into the service; and another, was a warrant-officer, advanced in years, who had served in harbour for several years. Deducting 10,000 for cases of gun-shot wounds, accidents, &c. the average number of cases of stone was then only *one* in 10,750. Now, Dr. Marcet states "the proportion of stone-cases received into the different British and continental public hospitals, Norwich alone excepted, to be *one* for every *three or four hundred* patients of all descriptions admitted. In the Norwich Hospital, it appears to be *one* in every *thirty-eight* cases." In this calculation, it must not be forgotten that, in the civil hospitals, the patients are of both sexes; and it appears, from Dr. Marcet's report of the Norwich Hospital, that the proportion of females operated upon is to that of males as *one* to *seventeen*: but here we must also remember, that females much more frequently void small stones through the urethra than men; and, therefore, the calculation of the proportion of the operation does not show the ratio of the occurrence of the disease in the two sexes.

The notion may here be entertained, that seamen may have frequently become invalided when affected with stone, and that this consideration should modify the application of the above statement. This circumstance has not been neglected by Mr. Hutchison; and the results of his enquiries have served to render the peculiar freedom of seamen from calculous disorders more evident. The general official reply, it is true, was, that no records were preserved of the diseases for which seamen had been invalided; but the surgeons positively asserted, that no instance had occurred at the royal hospitals of patients, so affected, being invalided previously to having undergone the operation of lithotomy.

The next step was, to determine whether or not many of the patients treated at the civil hospitals had been seamen; and here, too, the evidence tended to establish the same fact. Sir Everard Home and Mr. Cline stated, that, to the best of their recollection, they had never performed the operation of lithotomy on a seaman; and Mr. Astley Cooper said, that, to the best of his knowledge, only one instance of the disease alluded to in that class of persons had occurred to his observation in the course of his practice, and this was in Admiral B. D—s. He was not operated on: the stone, however, was ascertained to be of that species called mulberry, by a portion being broken off by the sound, and passed by the urethra. Mr. A. Cooper was consulted on this case a few years since; and Mr. Hutchison has since ascertained that the patient had not been employed at sea for the last twenty years of his life. In the public dispensary at Sunderland, no operation for the stone had been performed during the twelve years that Dr. Armstrong was physician to that institution; nor does that physician recollect having been once consulted by a sea-faring person affected with calculus. Dr. M'Leod states, that ten operations for the stone had taken place at Aberdeen in the last five years, but none of the cases occurred in a seamen. Aberdeen is the principal port of the north of Scotland; but Dr. M'Leod cannot call to mind any instance of a mariner applying for relief for the disease in question, during the time of his attendance there. The report of Dr. Ramsay, of Newcastle-upon-Tyne Infirmary, furnishes similar evidence. In between five and six hundred cases of operation for the stone at the Norwich Infirmary, occurring since its establishment in 1772, not a single instance of a mariner being the subject of it, occurred to the recollection of Dr. Rigby. Mr. Hardy, many years resident-apothecary in the same hospital, makes the same remark. Mr. Smith, one of the surgeons of the Bristol Infirmary, is engaged in some researches on calculous disorders, and furnishes some powerful evidence of the same character: and, with respect to the practice of the Infirmary, he says, "it has now been established eighty-three years, during the whole of which period there *is no stone case marked 'mariner;'* which it would have been, had a sailor applied to the recommender. There has been no sea-faring man out within my remembrance, which amounts to thirty-one years; and your own [addressing Mr. BAYNTON] recollection will carry you ten years above that."

If we compare these statements with the diet used by seamen, we shall have evidence elicited that powerfully controverts the hypothesis of the formation of urinary calculi being so much dependant on azoted aliment; for seamen are, Mr. Hutchison says, unavoidably compelled to subsist, often for months successively, on salt beef and pork; although, on the ship's arrival in port, the men are amply supplied with fresh beef, vegetables, and table-beer, while victualling and watering, which however, in time of war, is generally limited to a very short period. An observation, however, follows, which seems worthy of particular notice: it has appeared so to the author. Mr. Hutchison says, on their arrival into port, "it is incredible to see what quantities of salt seamen will use with their fresh beef." On connecting this with the ensuing paragraph, we have some views opened to us, perhaps intimately connected with the freedom of seamen from the disease under consideration.

"The beef or pork commonly issued to the ship's company at sea is so highly salted, and frequently kept so long in its briny pickle, that its bland and nutritious juices are in great measure exhausted. Excepting in ships of war of the first and second rate, a portion of one deck only is appropriated to the whole ship's company to sleep in, and this is consequently so crowded with hammocks, and the men so impacted together, (fourteen inches in width being the total space allowed to each individual,) that some dexterity is requisite to obtain ingress and egress to and from their beds. The lower deck being always the part allotted for repose, the ports are, for the safety of the ship, necessarily closed all night; and the temperature of the surrounding air is thereby so exalted, that the place becomes a kind of steam-bath from animal exhalation, the men being literally immersed in their own perspiration."*

In his reflections on those circumstances, Mr. Hutchison says,

"From Dr. Marcet's and Dr. Prout's remarks, it would appear that an active and healthy state of the digestive organs is one of the most effectual preventives against the formation of calculi. May it not therefore happen, in the instance of sea-faring men, that the peculiarities of their regimen, and especially the great quantities of muriate of soda they habitually take with their food, contribute to produce this effect?† or, in other words, shall we be justified in imputing to the

---

* "See Sir Gilbert Blane's excellent paper on the *Comparative Health of the Navy*, published in the sixth volume of the Transactions of this Society; and also some *Practical Observations in Surgery*, by A. Copland Hutchison, published in 1816, pp. 77 and 78."

† "See Dr. Marcet's Essay, p. 176; and Dr. Prout's valuable paper in the eighth volume of the Transactions of this Society, pp. 543, 544, 545."

stimulus communicated to, and maintained in, the whole chylopoietic viscera by the muriate of soda, a power to counteract the aggregation of calculous matter in the urinary organs, independently of any direct chemical agency?"

Dr. Marcet, and especially Dr. Prout, acknowledge that the measures they have seen used in the treatment of calculous disorders, have appeared, in most of the cases where they have been productive of benefit, to effect this rather by their restoring the healthy state of the digestive system, than by chemical agency. On a former occasion (vol. xli. pp. 110, 111,) when offering some objections to the hypothesis of M. MAGENDIE, we noticed how unsatisfactory many of his statements appeared, and totally inexplicable on chemical principles.

Mr. Hutchison lays some stress on the copious cutaneous perspiration elicited in seamen, in the state described in the paragraph last transcribed, in connexion with the query of Dr. Marcet, "whether there may not be some essential connexion between the state of the cutaneous functions, and the greater or less prevalence of this class of disorders."

The following observations, too, are particularly interesting:

"The life of a seaman (says Mr. Hutchison) is one of great activity, and often of considerable labour and exertion. I have frequently observed, in common with other officers, that sailors never fail to empty the bladder on the first symptoms of distention; and the facilities afforded them, as far as regards unmixed society and locality, favour greatly this salutary habit. It is also of importance to notice, that no description of people are less subject to dyspepsia,* or more prone to strictures in the urethra."

On the contrary, people of sedentary habits, the author continues to remark, especially lawyers and other studious men, and who are in habits of retaining the urine, are said to be more subject to the disease in question, than those devoted to the more active scenes of life, and greater muscular exertion.

Mr. Hutchison adduces several other observations favouring those views. He also notices the apparent influence of acescent fluids, such as cyder, malt liquors, and French wines, in favouring the generation of lithic gravel: but this we are disposed to attribute to their disordering the digestive functions, rather than to any immediate chemical agency on the remote parts of the system. Mr. Hutchison, however, in the following passage, advances a notion similar to that formed by M. Magendie: when speaking of the probable influence of sea provisions, he says,

"To acquire this prophylactic property, it may be essential that the animal food should be saturated with salt previously to its use; as

---

* See various parts of Murray Forbes's work on *Gravel and Gout.*

we learn from Dr. Wollaston, that, when free from saline matter, animal food favours the generation of lithic acid, at least in carnivorous birds.* Whether similar effects follow its application to the human stomach, has not yet been ascertained, I believe; but, reasoning from analogy, we might be induced to conclude, that such would be the consequence."

It would, however, be admitting more than we are well authorized to do, to state this positively; especially when we consider that remedies of opposite chemical qualities, or vegetable substances which cannot be supposed to act chemically at all, will commonly be beneficial in individual cases, when they relieve gastric disorder. We must not forget, too, that BERZELIUS states, as we some time since remarked, that the blood of herbivorous animals contains a greater proportion of azote than that of carnivorous. However, animal food may favour the production of lithic acid, from other reasons than because it contains so large a proportion of azote,—a circumstance which seems to have escaped the attention of M. Magendie; and therefore, his conclusions with respect to the influence of that species of food may be correct, although the arguments on which they were founded may be erroneous.

With this highly valuable paper we shall terminate the consideration of this volume: the *further observations on the proximate principles of the urine*, by Dr. PROUT, will be noticed in the general history of discoveries in animal chemistry, to be produced at the termination of the present year. We must, however, add our voice to the expressions of gratitude with which the labours of this Society have been universally received, and express our pleasure at seeing in this, another volume contributed to the classical medical literature of our nation.

---

*Observations on the Inflammatory Endemic incidental to Strangers in the West Indies from temperate Climates, commonly called the Yellow Fever, as this Disease occurred to the Author during a Public Service of Twenty Years in a majority of the West-India Colonies; with Notes and Illustrations. To which is added, an Appendix, containing Abstracts of Official Reports upon West-India Fevers, addressed to the Head of the Army Medical Department.* By NODES DICKINSON, of the College of Surgeons; Staff-Surgeon to H. M. Forces; Member of the Medical and Chirurgical Society, &c. 8vo. pp. 216. London: Callow, Underwood, and Burgess and Hill. 1819.

THE author of this work has so forcibly shown the errors and inconsistencies into which those persons have fallen, who have ventured to advance opinions on the subjects of it, founded on

* See *Philosophical Transactions* for 1810, p. 229.

any other basis than original observation, that we should be deterred from imitating their conduct, even had we not been before penetrated with the truth contained in his remarks. The impropriety of this conduct would, indeed, be soon detected, when applied to less speculative subjects than medical theory. Let a chemist attempt to apply new principles to the explanation of any series of chemical phenomena, merely from the ideas he can acquire from the results of experiments of others, he will be arrested at the onset;—be led to discern, that men in general see little more than those parts of objects which fall in with their peculiar views; and that original investigations will be necessary, to furnish a solid basis for an original train of reasoning.

The only critical remarks we shall, then, adduce on this work, are, that the author reasons with precision and apparent solidity of judgment, on the ideas acquired from his own observations; and that, in considering yellow-fever as an endemic malady originating from atmospheric influence, he is supported by some other experienced physicians of eminent talents: we would especially designate Dr. Veitch and Dr. Mac Arthur.

The precise disease that should be considered as yellow-fever, is a question yet disputed. Here we witness so remarkable an instance of the confusion which may ensue from the use of inappropriate names for diseases, that it has been chosen as an express subject for the illustration of this fact by Sir Gilbert Blane, in his "Elements of Medical Logick." *Typhus*, fever from *marsh miasma*, and the *atmospheric endemic*, are all commonly accompanied with a *yellow* colour of the skin; and hence much of the disputation on the subject of *yellow-fever* has obviously arisen. Each of the diseases above designated, is still termed yellow-fever by different physicians of eminence; and hence the partizans of animal contagion, marsh effluvium, and atmospheric influence, respectively argue on their own grounds with the certainty of gaining a triumph: whilst, to add to our embarrassment, the pathology of those diseases appears to be not sufficiently accurate to furnish a basis for precise distinctions between them.

Mr. Dickinson commences his work with stating, that extensive sources of observation, and long experience in the West-India districts, have led him to form an opinion that *yellow-fever* should be distinguished from *idiopathic* fever, whether resulting from animal contagion or from marsh effluvium. He was particularly led to form this distinction, "by observing the great number of patients who fell victims of yellow-fever in the year 1796, when all *but* new comers enjoyed complete exemption; and by having the opportunity, soon after, of contrasting the disease with the marsh remittent fever."

Several cases of the inflammatory endemic, the marsh remittent, and of a topical affection, marked, equally with the others, by yellowness of the skin, occurred at the same time, in the Military Hospital, under the author's superintendency; and, he continues,

"As these *distinct* febrile affections were each regarded as examples of the yellow-fever, from the predominancy of a striking symptom, (which, upon this occasion, led to an enquiry of Mr. Dickinson, by medical officers lately arrived in the West Indies, as to the identity of the diseases in the hospital with the yellow-fever of authors;) the importance was suggested of distinguishing them, by a more minute attention to their nature and cause; as, notwithstanding their resemblance in certain particulars, they require a different consideration both for their prevention and cure."

After stating the opinions that have been entertained respecting the origin of yellow-fever, from animal contagion, and marsh effluvium; acknowledging the occurrence of a form of fever from the latter source; and expressing doubts respecting the propagation of contagion in tropical climates; he observes,

"There is, however, a form of febrile affection, occurring so frequently and so extensively,—sometimes so violent in its symptoms, and so fatal in its results, as to excite surprise that it has not received attention proportionate to its importance.

"This febrile affection is the effect of sudden change of climate, augmented by violent exercise and intemperance.

"It is this disease which the writer denominates the 'inflammatory endemic of new comers to the West Indies from temperate climates.' Occurring less often, and in a comparatively slight degree, it has occasionally been called the 'bilious continued,' or 'sporadic fever.' In severe examples it has acquired the name of 'yellow-fever,' 'malignant,' 'pestilential,' &c."

This form of fever the author considers as dissimilar to the "idiopathic fever of human contagion; or of febrific miasma, the product of particular soils. Nor can it be regarded as a topical phlegmasia; for the general affection of the system precedes the topical determinations, which are *liable* to take place to certain organs:" and thus he thinks "the *yellow-fever* must not only be distinguished from contagious and marsh fever, but also from fever, as understood by those who refer it to the morbid affection of an individual organ."

An inflammatory diathesis is, then, Mr. Dickinson continues, invariably a predisponent cause of yellow-fever; and a state of *general excitement*, the chief characteristic of the disease: now, these circumstances, he concludes, are not essential to idiopathic fever.

This exposition would have been more clear and precise, if the author had first shown what he considered to constitute idiopathic fever; because, by designating this as not consisting in *general excitement*, nor dependant on local inflammation, he leaves us without a precise object for contemplation.

"The disease, (the author afterwards observes,) being exclusively incidental to strangers from temperate regions, will be found to occur with a prevalency proportioned to their numbers: sporadically, when these are few; or epidemically, at least in appearance, when many are introduced at the same time.

"Happening in a mild way, it may with great propriety be termed a *seasoning*. The reduction of the system by the evacuations necessarily employed for its removal, is commonly preventive of a future attack."

Long residence will produce the same effects; and what forcibly illustrates the causes of the particular disposition to this fever, is the fact, that natives of the country, and Africans, are exempt from it while they continue in the torrid zone, though, after a residence in a temperate climate, they acquire a susceptibility to it. The age of adolescence is that in which its attacks are most severe; and the natives of the colder regions, those who are most readily liable to it.

Some remarks follow, of very considerable interest, and seem to explain in a satisfactory manner occurrences that have been thought, under diversity of circumstances, to favour the doctrine of imported contagion, and the origin of the disease from marsh effluvium.

"The freedom of the West-India islands, at different times, from yellow-fever, has been ascribed to certain changes in the atmosphere, independently of the temperature; while the exemption of troops, *accustomed* to the climate, has been attributed to the clearing away woods, draining morasses, and erecting new barracks in more healthy situations.

"This occasional immunity, agreeably to the writer's observation, should, however, have been referred to the absence of susceptible subjects: for, whenever great numbers of strangers have recently arrived at the same time, the disease has usually appeared like an epidemic of awful extent; although the natives, negroes, and the other classes of the community assimilated to the climate, continued healthy."

But, at the same time with this affection, as before observed, fever from marsh miasma, and from local inflammation, sometimes occurs; and various terms have been employed to designate the particular affection, whilst they have retained the general appellation of yellow-fever.

As witnessed by Mr. Dickinson, the inflammatory endemic was invariably, he says, divested of the character of contagion. Its relations to idiopathic fever, as considered by Pringle, Cullen, Cleghorn, Lind, Fordyce, Currie, or their numerous followers, then become the subject of the author's remarks. The appearance of oppression or debility in the vital powers, mentioned by those writers as occurring at the commencement of simple idiopathic fever, was not witnessed in the inflammatory endemic: its earliest symptoms "consist in an increased excitement of the sanguiferous system; not as an accident, or irregularity arising in the course of the disease, but as an essential part of the attack, which well-conducted evacuations can alone control." The author continues to observe, that

"The inflammatory endemic, which, in its mildest form, has been regarded as a 'sporadic febricula' of ephemeral duration, has, under a severer aspect, when attended with yellowness of the skin, black vomiting, &c. been considered an infectious epidemic, or a malignant typhus."

After some general remarks on its changes of character in different stages, and the influence of treatment on its first attack in modifying them, the author continues his comparison with idiopathic fever. The symptoms of the first stage of the latter have no existence in the yellow-fever. "Many of those appearances which form the second stage, are amongst the primary symptoms of this disease; from the commencement of which, there is always increased velocity of the circulation, accompanied, in severe and neglected cases, by topical determinations to important organs." If this state of excitement be not subdued, a stage of debility will ensue, destructive to the patient. There are no renewal of febrile paroxysms, or remissions and exacerbations: "the appearances which afford hopes of a remission to those who are unprepared by experience, and which have often led to the exhibition of the cinchona, in the view of preventing a recurrence of the febrile stage, invariably tend to the fatal termination of the disease." Those appearances depend on extreme debility from exhaustion by the previous excitement, accompanied with some organic derangement.

Mr. Dickinson then adduces a comparison between this fever and the "marsh intermittent and remittent;" and with this terminates the introduction to the work.

The view we have here given of the author's general statement of his opinions respecting the origin and nature of the inflammatory endemic fever, is, we perceive, very imperfect, and somewhat of an indeterminate character. This, we must observe, has depended in a great measure on the construction of the work, of which we shall here give a concise explanation. That part of it comprising the author's own observations and reflections, was written as early as the year 1813; but, the publication having been delayed, notes and illustrations have since been added, which, continually breaking the train of the author's reasoning, render it almost impossible to proceed in it with that precision and order necessary to enable us to give a connected or adequate view of it, in so concise an abstract as our limits confine us to; though this is an inconvenience that will be of but little moment in the perusal of the work in detail,

The other divisions of it are devoted to the consideration of the *causes and prevention*, and the *symptoms and treatment*, of the yellow-fever: each of which are also accompanied with "notes and illustrations," comprising a further development of the author's opinions on several points, and extracts from the works of other writers.

We pass over the former, since, from the view already given, the author's general observations on the causes, and prophylactic measures, of this disease, must be sufficiently evident: for particular points, we refer the reader to the original. From the latter section we shall adduce a few extracts, in order to render the account of the disease which we have given, somewhat more clear and comprehensive.

After some remarks on the variation in the character of this form of fever in persons of different habits of body, and from the different degrees of intenseness and duration of its exciting causes, Mr. Dickinson observes,

"A slight attack has seldom been recognized to bear any strict affinity to the much-dreaded 'yellow-fever.' Considered merely as a 'seasoning,' it has rarely been regarded of the same kind; as produced by the same causes; and prevented, or removed, by the same general means which are applicable to that fever in its severer aspects.

"In a somewhat greater degree, this 'seasoning' has received the title of fever, a 'sporadic,' 'an unusual and slight occurrence.' In its more formidable character, conformably with the violence of the symptoms excited, it has been contemplated with great alarm. It has been considered a disease '*sui generis*,' sometimes identified with the most fatal of those idiopathic fevers, with which the observer may have been acquainted.

"These states of the inflammatory endemic present, however, only different aspects of the same disease; the diversity of appearances, with their ultimate effects, solely arising from their greater or less degree of violence. It should be, however, explicitly understood, that the termination of inflammation in the exhaustion of the vital power, constitutes, in the latter stage of the disease, a change in which, in the treatment, we must regard *degree* as *kind;* inasmuch as the remedy of the first stage would prove destructive in the second.

"In the less severe examples, exposure to the exciting causes is soon accompanied by heat and dryness of the skin, with increased action of the sanguiferous system. These effects will, however, commonly subside by rest, and removal from the further influence of this cause.

"In a more decided attack, the sensation of heat upon the surface is increased to a painful degree; but feelings of heat and slight chilliness alternate for a time, until the morbid temperature be perfectly established. This sensation of heat is accompanied by a general blush, tension, and dryness of the skin, flushing of the face, and inflammatory appearance of the eyes. There is a universal sense of soreness, flying pains, diminution of the power of muscular exertion, restlessness, sighing, yawning, and stretching; occasionally a disposition to doze, but this disposition is not followed by tranquil sleep.

"These symptoms commonly precede, and accompany, a greater or less degree of pulsating pain within the head, particularly severe over the eyes, and rendered more so by pressure on the eye-balls. Pain is felt in the course of the spine, across the loins, and in the lower extremities. There is increased sensibility to the impression of light and sound. If any nourishment be attempted to be taken, it produces nausea. The tongue is clean, red, dry, and hot; sometimes white, with heat and dryness. Thirst is generally urgent; the pulse is commonly full and frequent, amounting to 130 in a minute. The respiration is laborious, with a painful sense of stricture across the chest. The bowels are costive; sometimes there is vomiting very early in the attack, with singultus, and pain, on pressure, at the pit of the stomach. The urine is scanty and high-coloured: its evacuation is attended with difficulty, and with scalding pain."

In more severe attacks, or when the disease proceeds uncontrolled, the author describes it as having the character ordinarily considered proper to yellow-fever. We cannot follow him through this history: but we may remark, that the principal subsequent phenomena, when determination to the liver takes place, are, yellowness of the skin, if the passage of bile to the intestines be interrupted; or, if this be free, vomiting and purging of dark bilious matter, afterwards assuming a tar-like appearance, and at length that of the *black vomit.* Determinations to other organs also supervene, especially to the stomach and brain; and, finally, hæmorrhages from various parts, and all the appearances commonly termed *typhoid.*

We adduce the following extract, to give a view of the author's general precepts for the mode of treatment:

"The consideration of the stages of increased action, and finally of exhaustion, determines the rationale of the treatment; as an attention to the producing causes, predisponent and exciting, afforded the ground of prevention.

"If, then, at the moment of the attack, the stomach should be loaded with solid matter, or over-stimulated by strong drink, an emetic will remove the impression of this exciting cause.

"Every other occasion of increasing the excitement must be obviated: after which, 1, general bleeding; 2, active purging; 3, the warm bath; 4, cold ablution, and cool drink. These must be employed to reduce morbid action, and prevent the debility resulting from over-excition generally, and from over-distention of particular vessels, causing congestion; while, in the actual occurrence of determination of blood to the head, stomach, or liver, topical bleeding, blisters, &c. must be resorted to, with the view to remove congestions already formed, and allow the vessels to recover their tone.

"If, however, exhaustion has supervened, the practitioner can administer but feeble aid. Quietude, a cool temperature of the atmosphere, gentle laxatives, nourishment, and sleep, present the only means of restoration.

"Nor will the most assiduous application of cordials, stimulants, anodynes, or antispasmodics, as certain medicines are sometimes called, often reward the anxious hope of the practitioner, even when aided by the more efficient agency of blisters, injections, and opium; although it will be proper to give them judicious trial."

Dissection generally showed signs of inflammation of the brain; in the stomach, a sort of pellicle, easily removed, covering the villous coat; and dark-coloured spots, apparently from effused venous blood, dispersed over the same surface. When symptoms of determination to the liver had been present, that organ was sometimes found to be livid and overspread with dark-coloured spots throughout its structure, much enlarged, and full of dark-coloured blood, or deep-yellow bile. The gall-bladder and hepatic ducts were occasionally tinged with black viscid bile.

A desire to furnish the generality of our readers with something like a view of the observations and opinions of Mr. Dickinson, has led us to make the foregoing abstract: it will, probably, be sufficiently comprehensive for the use of the civil practitioner; but men called to witness scenes similar to those in which the author for so long a period employed his useful exertions, will think it necessary for them to study in detail the results of the experience of so accurate an observer.

---

*Reports on the Diseases of London, and the State of the Weather, from* 1804 *to* 1816: *including Practical Remarks on the Causes and Treatment of the former; and preceded by a Historical View of the State of Health and Disease in the Metropolis of past Times; in which the Progress of the extraordinary improvement in Salubrity which it has undergone, the Changes in the Character of the Seasons in this respect, and the Causes of these, are traced down to the present Period.* By THOMAS BATEMAN, M D. F.L.S. &c. Physician of the Public Dispensary, and Consulting Physician to the Fever Institution in London. 8vo. pp. 288. London: Longman and Co. 1819.

THE title of this work sufficiently indicates the objects the author had in view in its production; and those who have perused the Reports of Dr. JOHN REID, published in the Monthly Magazine; and those cf Dr. WILLAN, in some of the early volumes of this Journal, need not be informed how interesting and useful one of the series of subjects it comprises may be made, by a person who has the ability to deduce general principles from his own observations; and, in descending to more particular views of disease and the effects of remedies, to select those points which are essential and of real importance, from what are common and accidental. The Reports of Dr. BATEMAN bear this character in a strongly-marked degree; and though, from the source of his observations, it is to London practitioners that they must be particularly interesting, yet it will be found that there are few diseases respecting which he has not advanced more or less numerous and original remarks, of general as well as of topical application and utility.

The author's historical account is, we venture to assert, superior to any thing of the kind in existence. His reasonings bear the mark of sound judgment, and they are derived from accurate and comprehensive views. Nothing of importance, indeed, at all connected with the subject, seems to have escaped him; and his production is as interesting to the reflective physician, as it will be found valuable to the political historian.

We transcribe the following passage, rather as a specimen of the author's manner, than because it possesses peculiar merit, compared with the rest of the dissertation:

"It will be easy, we apprehend, to show, in the first place, that the circumstances to which the diseases and mortality of the seventeenth century were attributed by the writers of those times, were not the real source of the mischief; and, secondly, that others, which were then almost entirely overlooked, were in reality the efficient causes of all the prevailing maladies.

"The coldness and vicissitudes of weather, and the humid and thick air, which were ascribed partly to our insular situation, and partly to the extensive use of sea-coal as fuel, are the chief circumstances to which writers have referred the origin of so much disease. Now, with respect to the first point, although we may question the truth of the opinion that our climate has of late years become more ungenial, we have no reason to believe that our seasons have become milder, or are in any way improved. With respect to the inclemency of our spring, we may quote Claremont, who wrote in 1652: 'Ac mihi nunc ista mense Maio scribendi, (he says,) videnter brumales dies; usque adeo denso et caliginoso aëre circumfundor;'*—an observation applicable to the same month for several years back. And I shall presently attempt to show, that the sickliness of the autumnal season did not arise from the difference in the season itself, but of the circumstances of the metropolis at the period alluded to.

"The contamination of the atmosphere of London by the smoke from coal-fires, appears to have been generally considered, both by natives and foreigners, as a leading cause of the unhealthiness of the town. 'I am inclined to believe,' says Major Graunt, 'that London now (1662) is more unhealthful than heretofore; partly for that it is more populous, but chiefly because I have heard, that, sixty years ago, few sea-coals were burnt in London, which are now universally used. For I have heard that Newcastle is more unhealthful than other places, and that many people cannot at all endure the smoke of London, not only for its unpleasantness, but for the suffocations which it causes.'† Evelyn, who published a pamphlet entitled *Fumifugium* in the year 1661, exclaims very feelingly upon this subject, 'that this glorious and ancient city, which from wood might be rendered brick, and, like another Rome,

---

* Claromontius, *de Aëre, Locis, et Aquis Terræ Angliæ*, p. 18.

† Major Graunt's *Natural and Political Obs. on the Bills of Mortality*, p. 95.

from brick made stone and marble; which commands the proud ocean to the Indies, and reaches to the farthest Antipodes; should wrap her stately head in clouds of smoke and sulphur, so full of stink and darkness, I deplore with just indignation.' And he states a curious fact respecting the temporary disuse of coals, as influencing vegetable life, from which its effects on animal life are to be inferred.* In refutation of these notions, however, it is sufficient to observe, that the supposed effects have ceased, while the cause has been augmented to an incalculable degree. And a similar observation will serve to refute an opinion, generally advanced by foreigners in particular, that most of the diseases of England are to be ascribed to the use of animal food and malt-liquors, especially taken to repletion, as was then the custom in this country, if we credit their report.†

"But the real sources of the unhealthiness of London, at the period when Willis and Sydenham wrote, eluded the observation of those sagacious enquirers, and remained for development by the gradual experience of a more enlightened and scientific age; although, perhaps, that experience has been, in a great measure, the unforeseen result of the necessities of increasing commerce, and of the contrivances of increasing wealth and civilization. This subject may be illustrated by a reference to the condition of an army in camp. The diseases by which London, in common with all large towns, was almost constantly infested, during and previous to the seventeenth century, were, as we have seen, the plague, malignant, intermittent, and remittent fevers, and dysentery. Now, these very diseases, according to the concurring testimony of all military physicians, are the regular endemics of camps, especially in the autumnal season, if they continue for a short time stationary, or are situated on damp or swampy ground. These diseases are obviously occasioned by the miasmata arising from a soil naturally wet, or rendered so by the accumulating filth of an army; such as the urine and excrements, the remains of victuals, the water used in cookery or in washing, &c. which is necessarily impregnated with animal and vegetable matter; and from all of which those morbific effluvia arise, in consequence of the putrefaction and more ready evaporation produced by the heats of autumn. This is not now a matter of speculation; for experience has amply proved, that, by draining a small marsh, by removing a camp even a few hundred

* In the year 1644, when Newcastle was besieged, and coals not to be procured in London, he affirms, that "divers gardens and orchards, planted in the very heart of London, (as, in particular, my Lord Marquisse of Hertford's, in the Strand, and my Lord Bridgewater's, and some others, about Barbican,) were observed to bear such plentiful and infinite quantities of fruit, as they never produced the like, either before or since, to their great astonishment."

† It is matter of curious remark, at what an early period the English had obtained the character of being greater eaters than their neighbours. We not only find Claremont speaking of the "cibi crassi ac fæculentæ turbidæque potiones" of our ancestors in the seventeenth century, and asserting that this diet was "non tam qualitate quam copia et quantitate insalubre," and that *repletion* was, in his opinion, the cause of all the diseases of the English, (see pp. 49, 51, 53;) but, so early as the latter end of the fifteenth century, that opinion existed abroad. For Cogan, in his *Haven of Health*, published in 1605, quotes Hadrianus Barlandus; who, in a dialogue between an innkeeper and a traveller, speaks of a rich and copious repast, as being "after the English fashion." See his prefatory address "to the gentle reader."

yards from the station which it previously occupied, by burying the excrements, and forming proper receptacles under ground for the urine, the water employed for culinary and other purposes, the remnants of victuals, &c. those endemic diseases have been removed, or altogether avoided.*

"Now, a large town is but an extensive camp, so constructed as to be destitute of the means of changing its situation, and therefore liable to be infested with the same diseases as are endemic in camps, unless the precautions just alluded to be fully adopted. Hence the necessity for the construction of privies, drains, and common sewers; and the advantages of a flowing stream, by which all impurities may be carried off; as well as of an abundant supply of water, for the purposes of cleanliness; and of a hard and regular pavement, preserved in a cleanly condition by proper scavengers, &c. in every crowded town. Wherever these precautions are neglected, intermittent and remittent fevers, and dysentery, will not fail to appear, especially in or after wet and hot seasons:† for, in the wet seasons, collections of filth accumulate; and, in great heats, they more readily putrefy and evaporate. Dr. Hunter expresses a strong suspicion, that the fatal endemic fever of Philadelphia, in 1793, was the result of neglect with regard to those precautions, as well as the subsequent endemics of New-York.‡

"It will not, however, be difficult, we apprehend, to prove, from documents in our possession, that those precautions of cleanliness, which alone are adequate to the removal of the sources of the miasms that excite endemic diseases, were not sufficiently attended to in London before the great fire in 1666, nor till upwards of half a century subsequent to that calamitous, though ultimately beneficial, event. And, what is not less convincing, as a proof of the pernicious consequences of such inattention, we shall find the health of the inhabitants improving, *pari passu*, and exactly in proportion as these causes of their unhealthiness were removed; while the damps and clouds of spring returned as heretofore, and the rains and heats of autumn continued."

---

***Researches into the Nature and Causes of Epilepsy, as connected with the Physiology of Animal Life and Muscular Motion; with Cases illustrative of a new and successful Method of Treatment.*** By John G. Mansford, Member of the Royal College of Surgeons of London; Author of an "Inquiry into the Influence of Situation on Pulmonary Consumption," &c. 8vo. pp. 160. Longman and Co. 1819.

THIS work is composed of two parts: one constituted of some hypothetical speculations on the causes of life; the other containing some observations respecting the treatment of epilepsy by the application of the principles regulating galvanic phenomena. The former we should pass over unnoticed, did we not think it our duty to censure the spirit by which it is

---

* *Obs. on the Diseases of the Army in Jamaica*, by Dr. John Hunter, Appendix, 3d edit.

† *Obs. on the Diseases of the Army*, by Sir John Pringle, part ii. chap. i.

‡ Dr. Hunter, *loc. cit.*

characterized. Mr. Mansford adduces the definitions of life given by some of those physiologists who think that this should be drawn from the description of its phenomena; and then, mistaking, apparently, those descriptions of results for propositions of primitive causes, he attempts to show the absolute erroneousness of the whole of them, and applies to the sentiments of their authors the most odious of appellations; saying, "on whatever ground materialism and infidelity (for they are inseparable) rear their imposing fronts, on that ground they must be met."

By what intellectual operation the conclusion is formed, that the authors of several of those definitions are materialists, we know not; but it is certainly not by correct reasoning: and what Mr. Mansford means by *infidelity*, we do not precisely comprehend; but, as this term is commonly considered to designate *atheism*, it becomes the imperative duty of every medical physiologist to express his disgust at such conduct as that we have just noticed. For, what must the public (whom, it is obvious, Mr. Mansford supposes will read his book) think of a profession generally, if such an accusation of the character of some of the most eminent and most esteemed of its members, be suffered to pass without reprehension? All those, then, who have a due respect for their own moral as well as scientific character, will vindicate themselves by an exposure of the folly, as well as mis-statements, evinced in the work before us.

For medical readers, it is only necessary to transcribe the first of a series of conclusions formed by the author. He says, "The immaterial principle, or soul, is the first and *only* principle of life and action; from which, in a descending series, *all* the motions of the living body must proceed, and which only can be entitled to be called the principle of life."

On this we shall make no comment. We have too much respect for the intelligence of our readers, to permit us to adduce a single reason in opposition to it; and the only remarks we choose to make respecting the arguments which lead to it, are, that any person who may determine to peruse them, must prepare to have his feelings shocked by the uncandid, and often grossly false, manner in which the opinions of others are stated; as well as to find common sense outraged by what are given as trains of rational reflection. It is necessary to add to the above, that Mr. Mansford believes the electric matter, or at least an analogous fluid, to be the medium by which the soul acts on the organization of *man*. Whether or not he considers that it is by the same matter that the souls of *brute animals* act on their bodies, he does not mention. We can hardly doubt but that he believes brutes possess souls; or to what will he attribute

*all* their vital actions?—perhaps, to electrical influence alone, as he is disposed to do those of plants.

In order that neither Mr. Mansford's, nor our, meaning may be mistaken on this occasion, we should say, that by the *soul* Mr. Mansford does not signify the divine *aura*, which some of the wisest of the Greeks supposed to be diffused throughout the whole creation, giving life to plants as well as to animals;—a sentiment that has been so often cited from VIRGIL, with approbation, by some of our philosophical Christian divines, (who, of course, consider that it does not oppose the doctrine that man, in addition to that, possesses that peculiar immortal existence commonly understood by the soul,) and which LUCAN made so impressive in the voice of Cato. St. PAUL, indeed, is in exact conformity to the doctrine of the Greeks on this subject. He speaks both of the Πνεῦμα (the *soul*) and the Ψυχὴ (the *vital principle*), in addition to the Σῶμα (the *body*); but, let it be observed, neither, nor both, of these were supposed to constitute in themselves the *mind* or *intellect*. ANAXAGORAS first taught that the mind was created, in a manner, by means of the principles above enumerated, from impressions received from external objects: it was for this reason that the doctrines of THEOPHRASTUS, DEMOCRITUS, and PARMENIDES, which taught that the soul and the mind were identical, were considered to favour materialism. Here St. Paul also accords with the opinions of the philosophers first alluded to: on this point, see his *Epist. ad Ephes.* c. 4. v. 17; and, on the former, his *Epist. ad Thessalon.* l. i. c. 5. v. 23. Mr. Mansford means the *soul* designated by the dogmas of the *Scriptures;* which, he says, authorize the belief, that such a cause is the "*only principle of vitality.*"

We turn to the part of the work appropriated to practical observations; the detail of Mr. Mansford's hypothesis of the nature of epilepsy is passed over, because it is founded on the principles above designated. The following paragraph will, perhaps, give the reader a sufficient idea of it:

"The voluntary motions of the body may be defined to be the result of a subtile and mobile matter, answering in its nature and properties to the electric fluid transmitted by an act of the will from the brain to the muscles. In a state of health, the principle of life is fully competent to regulate the formation, and the retention or discharge, of this substance. But, if it be weakened by disease, so that it may be unable to control that portion with which the brain is already charged, or to prevent its increase, or transmit it to the distant parts, the balance between its formation and expenditure being destroyed, an accumulation must happen; which arriving at its maximum, the point beyond which the brain cannot be charged without injury to its structure or functions, or perhaps without endangering life itself, the vital principle being absolutely overwhelmed and losing its command, the motive powers of the system become for a time obedient to those laws which would govern them in any other situation, and fly from the points in redundance to those in deficiency; when the vital principle, being freed from the load which threatened its existence, resumes its seat and its power.

"This may be considered as a brief explanation of the phenomena of the epileptic paroxysm; rendered still more probable by its general periodical character, when existing in its pure idiopathic form."

The application of a medium, then, by which the galvanic or electric matter* may be constantly carried from the brain, by which, of course, its undue accumulation, and the consequent explosions, may be prevented, is the new measure proposed by Mr. Mansford in the treatment of epilepsy. As we are not disposed to doubt the validity of his testimony, when he states the results of his observations, we must observe, that the facts he adduces show his measures to be worthy of attentive consideration. Useful practical means have often resulted from erroneous reasonings: the administration of iron in chlorosis is a forcible illustration of this.

Several cases are related, that were relieved in a remarkable manner by Mr. Mansford's remedy, some of them chronic, others acute: and, what seems to show that these results did not depend on moral influence, is, that at least one of the patients was too stupid to be impressed in this way; and, in one instance particularly, a recurrence of attacks, after they had totally disappeared for a considerable period, took place at different times; when it was found, on all these occasions, that the conducting medium was broken.

Mr. Mansford does not mean that this measure should exclude the use of other moral and physical measures,—as blood-letting, purgatives, &c. according to the indications of symptoms; neither does he suppose that it will effect a cure when the disease depends on organic lesion of the brain; but even these cases, it appears from his observations, may be alleviated by it. One instance of what might be considered hereditary disease, was so much relieved, that nothing but the patient's obstinacy seemed to prevent the perfect success of the remedy.

Mr. Mansford's mode of applying galvanic influence to the cure of epilepsy, is a modification of that first used by Dr. Grapengiesser, of Berlin,† in palsy, and various other diseases. But that physician did not employ it with the view of constantly carrying off electric matter from the brain. He also applied the metals to the exposed cutis, which, Mr. Mansford says,—

* We use these terms as if we were confident of their propriety; but the doctrine of the existence of such a *matter* (not a *quality* of other matter) is shaking to its foundation. This was stated by Dr. Thomson, in his last History of the Progress of Chemistry, in the *Annals of Philosophy*.

† A full account of this is given in the eighth and ninth volumes of this Journal.

"Must have been intolerable to many patients for any length of time; besides that in an epileptic habit, which is generally an irritable one, it would not be admissible. Dr. G. further recommends, that the metals be loosely applied, that the connexion may be alternately closed and interrupted: but this is still worse; and, besides not answering the intention in view, I am confident that very few would bear this form of this remedy, and that in very many instances it would be highly injurious. It was obvious, then, that unless some means could be devised, by which the pain and irritation attending this method could he lessened, the object in view could not be obtained. The insertion of some soft conducting substance between the metals and the exposed surfaces suggested itself, as likely to obtain this desirable end; and, after many trials, a piece of moistened sponge was found to succeed, with the surface covered by a silver plate, and a bit of muscle freed from any portion of fat or cellular membrane, with that covered by the zinc. This difference was found necessary, from the different effect produced on the two surfaces: that communicating with the silver, affording a slight serosity, sufficient to keep the sponge moist, and to preserve the conducting power; while the surface in contact with the zinc did not afford sufficient moisture, and the intervening layer of sponge, in consequence, acted as a non-conductor. Many things were tried as substitutes for the bit of muscle; but this was the only substance found which could be depended on for maintaining a regularity of action for any length of time, without exciting uneasiness. By this arrangement, the principal difficulties which had at first presented themselves were surmounted; and it remained now only to adapt it to the purpose required, with as little inconvenience to the free motions of the body and the habits of life as possible.

"It was said, that, in order to fulfil the indication stated at the commencement of this section, it was desirable to establish a negative point as near the brain as possible, and a positive one, in some distant part of the body. Accordingly, a portion of the cuticle, of the size of a sixpence, being removed, by means of a small blister in the back of the neck, as close to the root of the hair as possible, and a similar portion in the hollow beneath, and on the inside of the knee, as the most convenient place: to the wound in the neck, a plate of silver, varying, according to the age of the patient, from the size of a sixpence to that of a half-crown, was applied; having affixed to its back part a handle or shank, and to its lower edge, and parallel with the shank, a small staple, to which the conducting wire was fastened. This wire descended the back till it reached a belt of chamois leather, buttoned round the waist; it then followed the course of the belt, to which it was attached, till it arrived opposite the groin on the side it was wished to be used; it then passed down the inside of the thigh, and was fastened to the zinc plate in the same manner as to the silver one. The apparatus so contrived was thus applied: A small bit of sponge moistened in water, and corresponding in size to the aperture in the neck, was first placed directly upon it; over this a larger bit of sponge, of the same size as the metallic plate, also wetted, was laid; and next to this the plate itself, which was secured in its situation by a strip of sticking-plaster* passed through the shank on its

* The plaster I have generally used consists of equal parts of lytharge and soap plasters, with the addition of a very small quantity of resin, to render it a little back, another above, and another below it. If these be properly place d and the wire which passes down the back be allowed sufficient room th a it may not drag, the plate will not be moved from its position by any ordinary motion of the body. The zinc plate was fastened in the same manner; but, in place of the second layer of sponge, a bit of muscle, answering in size to the zinc plate, was interposed: that is, a small bit of moistened sponge being first fitted to the aperture below the knee, the piece of muscle, also wetted, then followed; and on this the plate of zinc. The apparatus thus arranged will continue in gentle and uninterrupted action from twelve to twenty-four hours, according to circumstances. This last is the longest period that it can be allowed to go unremoved. The sores require cleaning and dressing, and the surface of the zinc becomes covered with a thick oxyde, which must be removed, to restore its freedom of action: this may be done by scraping or polishing. But it will be better if removed twice a-day, both for the greater security of a permanent action, and for the additional comfort of the patient."

For more particular directions on this subject, we refer the reader to the work.

The same motives that made us pass such unqualified censure on the contents of the first part of this book, lead us now to express our sentiments of approbation of that we have just alluded to. There is much ingenuity in the application of the author's remedy; and, if it proves as efficacious in the cure of epilepsy as he anticipates, he need desire no other claim to the esteem of the public, as well as of his professional brethren. The errors we pointed out at the commencement of his work, are such as mark all those not directed by the spirit that dictated the axiom with which one of our truly philosophic treatises on Pathology commences, "*All human science is a knowledge of the qualities and order of phenomena.*" With this we must be content in physiology; and, since the best-informed men in other branches of science contemn "the explicit assertion both of Moses and Solomon," and profess the Pythagorean, or the Copernican, as it is called, doctrine of the relation of the motion of our sun and the earth, we may be allowed in physiology to think, contrary to the opinion of Mr. Mansford, that their assertion on this subject does not demand our assent; and that some respect is due to those men who think proper to reason on the sensible phenomena of the human body, as they do on those of nature in general.

---

more adhesive. This plaster is clean in its appearance, and seldom irritates the skin by long use.

